# Combined Effects of High-Speed Railway Noise and Ground Vibrations on Annoyance

**DOI:** 10.3390/ijerph14080845

**Published:** 2017-07-27

**Authors:** Shigenori Yokoshima, Takashi Morihara, Tetsumi Sato, Takashi Yano

**Affiliations:** 1 Kanagawa Environmental Research Center, 1-3-39 Shinomiya, Hiratsuka, Kanagawa 254-0014, Japan; 2 Department of Architecture, National Institute of Technology, Ishikawa College, Kitachujo, Tsubata, Ishikawa 929-0392, Japan; morihara@ishikawa-nct.ac.jp; 3 Department of Architecture, Hokkai-Gakuen University, Nishi 11-1-1, Minami 26, Chuo-ku, Sapporo 064-0926, Japan; sato@arc.hokkai-s-u.ac.jp; 4 Professor Emeritus, Kumamoto University, Kurokami 2-39-1, Chuo-ku, Kumamoto 860-8555, Japan; yano@gpo.kumamoto-u.ac.jp

**Keywords:** high speed train, noise, ground vibration, social survey, secondary analysis, annoyance, exposure-annoyance relationship, logistic regression analysis

## Abstract

The Shinkansen super-express railway system in Japan has greatly increased its capacity and has expanded nationwide. However, many inhabitants in areas along the railways have been disturbed by noise and ground vibration from the trains. Additionally, the Shinkansen railway emits a higher level of ground vibration than conventional railways at the same noise level. These findings imply that building vibrations affect living environments as significantly as the associated noise. Therefore, it is imperative to quantify the effects of noise and vibration exposures on each annoyance under simultaneous exposure. We performed a secondary analysis using individual datasets of exposure and community response associated with Shinkansen railway noise and vibration. The data consisted of six socio-acoustic surveys, which were conducted separately over the last 20 years in Japan. Applying a logistic regression analysis to the datasets, we confirmed the combined effects of vibration/noise exposure on noise/vibration annoyance. Moreover, we proposed a representative relationship between noise and vibration exposures, and the prevalence of each annoyance associated with the Shinkansen railway.

## 1. Introduction

The Shinkansen super-express railway, the high-speed bullet train in Japan, has greatly increased its transportation capacity since the opening of the Tokaido Shinkansen line in 1964. Since then, the network of Shinkansen lines has been continuously expanded in Japan. The Hokuriku and Hokkaido Shinkansen lines were partially opened in 2015 and 2016, respectively. However, noise, ground vibration, and low-frequency sounds from operating trains continue to annoy inhabitants living in areas along the lines. To preserve living environments and protect inhabitants’ health, the Japanese government enacted the Environmental Quality Standards for Shinkansen Super-express Railway Noise, in 1975. The following year, the Director of the Environment Agency recommended that “Shinkansen railway vibration countermeasures be urgently undertaken for environmental preservation.” Enforcement of the environmental quality standards and recommendation has improved the noise and vibration in the areas along the lines; however, not many inhabitants remain disturbed by both the noise and ground vibration.

Yokoshima et al. [1] reported measurements of building vibrations induced by the Shinkansen railway. For vertical vibrations, Shinkansen railway-induced vibrations slightly increased when transmitted to wooden detached houses and decreased when transmitted to reinforced concrete apartment buildings. Thus, it is presumed that inhabitants living in detached houses are exposed to greater vibration than those in an apartment. Based on noise and vibration measurements in the areas along the Sanyo Shinkansen line and adjacent conventional railway lines, Yano et al. [2] showed that the Shinkansen railway emitted greater ground vibrations than the conventional line at the same noise level. Thus, it is likely that building vibrations affect living environments as significantly as the associated noise.

A number of social surveys on the community response to Shinkansen railway noise and vibration have been carried out in Japan. Yokoshima et al. [3] reported on temporal changes in the community response to noise and vibration from 1985 to 2002. Yano et al. [2] conducted a social survey and noise and vibration measurement along the Sanyo Shinkansen line and suggested the presence of an interaction between noise and vibration from the Shinkansen railway. Yokoshima et al. [1] found that annoyance due to noise and vibration from the Shinkansen railway increased with the associated vibration and noise in detached houses, not in apartments. Based on these results, they introduced the concept of “combined annoyance” due to noise and vibration, and clarified the equivalent effect of each exposure on the combined annoyance [4]. Furthermore, they proposed not only a noise exposure-response relationship [5,6] but also a vibration exposure-response relationship [7]. Following these studies, more recent results of new social surveys have been reported. Morihara et al. [8] investigated the exposure-annoyance relationship associated with noise and vibration due to the Nagano Shinkansen line. Using a logistic regression analysis, they indicated a relationship between the day-evening-night sound level (*L*_den_) and annoyance associated with the Nagano Shinkansen railway. The exposure-annoyance relationship was higher than that for the Miedema’s railway curve [9]. Tetsuya et al. [10] compared the exposure and annoyance associated with noise and vibration in areas along the Kyushu Shinkansen line and conventional railway line. A comparison of before and after the opening of the Kyushu Shinkansen line showed that high annoyance did not change significantly, but moderate annoyance decreased significantly following the opening. In contrast, there was no significant difference in either high or moderate annoyance between the periods before and after the opening of the conventional railway line. Apart from these studies, Yokoshima et al. [11] compared the exposure-response relationships for transportation noise using datasets accumulated in Japan and showed that the Shinkansen railway noise was equally as annoying as commercial aircraft noise and more annoying than conventional railway and road traffic noise. This finding indicates that there is no bonus to these railways in Japan, unlike European and North American countries. They reported that the difference between the absence and presence of a railway bonus could be attributed to the effect of associated house vibrations on noise annoyance.

In addition to surveys, a number of laboratory-based experiments have been performed to examine human response to high-speed trains. Lee et al. [12] investigated the effects of noise and vibration on annoyance in buildings during the passage of a nearby high-speed train with a recorded train noise and 20 Hz of vibration. They found that vibration did not influence the noise annoyance rating, but that the total annoyance caused by the combined noise and vibration was considerably greater than the annoyance caused by noise alone. Morihara et al. [13] carried out an experimental study to examine the combined effects of high-speed railway noise and vibrations on activity disturbances, using noise and vibrations from the Hokuriku Shinkansen railway as stimuli. The results indicated that there was a combined effect of noise and vibrations on disturbances in thinking and reading tasks at low levels of noise exposure.

The results of previous studies indicate that noise and vibration induced by the Shinkansen railway likely have a combined effect on annoyance. However, the relationship between both exposures and each individual annoyance has not been quantified. Therefore, to clarify the combined effect, we performed a secondary analysis using individual datasets of noise and vibration exposures, as well as community responses associated with the Shinkansen railway. Using data compiled through six socio-acoustic surveys conducted separately over the past 20 years in Japan, we compared exposure-response relationships associated with the Shinkansen railway noise and vibration by dataset. Then, we applied a logistic regression analysis to the datasets and examined the effect of noise and vibration exposures on annoyance. Finally, we adjusted for confounding factors to reveal and show the relationships of noise exposure, vibration exposure, and the related annoyance.

## 2. Methods 

### 2.1. Social Surveys

In this study, we examined data from four Shinkansen lines. The Tokaido Shinkansen line (TSL), which opened in 1964, currently operates more than 300 trains per day. The Sanyo Shinkansen line (SSL) began operation in 1972 and shares a track with the TSL. The Hokuriku Shinkansen line (HSL) was originally operated from Tokyo to Nagano in 1997 and reached Kanazawa in 2015. Kyushu Shinkansen line (KSL) began operation in 2011 and runs from Fukuoka to Kagoshima via Kumamoto.

The individual datasets analyzed in this paper were derived from six social surveys: Kanagawa (KNG95 and KNG01), Fukuoka (FKO), Nagoya (NGY), Kumamoto (KMM), and Nagano (NGN) surveys (see Table 1). Regarding annoyance due to Shinkansen railway noise (hereafter denoted as noise annoyance), five surveys used the International Commission on Biological Effects of Noise (ICBEN) verbal scale, where the possible responses included: 1. not at all; 2. slightly; 3. moderately; 4. very, and; 5. extremely. However, it should be noted that the NGY survey measured noise annoyance from the railway without specifying the Shinkansen line. In addition, the KNG95 survey measured annoyance only for the two most bothersome noise sources on a four-point scale (1. bearable to 4. unbearable). When Shinkansen railway noise was chosen as one of the most bothering sources, the four-point scale was used to refer to noise annoyance; otherwise, the noise annoyance was allotted to the “not at all bothered” response. Thus, noise annoyance in this survey could be converted into a five-point scale.

Annoyance due to ground vibration (hereafter denoted as vibration annoyance) was measured using the same scale as noise annoyance. However, for the NGY dataset, the vibration annoyance associated with building vibrations perceived in the dwelling was measured using the ICBEN scale.

The KNG01 and KMM surveys targeted inhabitants living in both detached houses (DH) and apartment buildings (AB). Noise and vibration exposures transmitted from the railway differed greatly between the two housing types, depending on insulation performance and foundation. Therefore, this study focused on the datasets of detached houses, and included 2967 responses.

### 2.2. Noise and Vibration Measurements

Measurement of noise and ground vibration from the Shinkansen railway was conducted on a site-by-site basis for each survey. For all of the included surveys except NGY, measurements were performed after the social survey was completed. Conversely, for the NGY dataset, the city government performed the measurements before the social survey. It should be noted that vibration exposure was not estimated for some inhabitants in the FKO survey.

Regarding the noise measurements, the maximum A-weighted and S-weighted sound pressure level (*L*_ASmax_) and the A-weighted sound exposure level (*L*_A*E*_) of the pass-by noise from the Shinkansen super-express trains were measured at several points at different distances from the track. At each point, the maximum-based and energy-based indices (*L*_Amax_ and *L*_dn_, respectively) were calculated as noise exposures. The former was calculated as the mean energy value of the top 50% of the measured *L*_ASmax_ values. The latter was calculated as the mean energy value of measured *L*_A*E*_ and the number of pass-by trains during the daytime (7:00–22:00) and nighttime (22:00–7:00). From the above results, depending on noise emission and propagation conditions, one or more distance attenuation equations were formulated for each noise index via logarithmic regression. Then, the noise exposure to each respondent was estimated based on the corresponding formula. 

Similarly, the maximum vibration level in the vertical direction (*L*_vzmax_) on the surface of the ground was measured at almost the same points as the noise measurements. The reference acceleration of the vibration level is 10^−5^ m/s^2^. The maximum-based index (*L*_vmax_) was calculated from the mean value of the top 50% of measured *L*_vzmax_ values. The vibration exposure for each respondent was estimated using the same method as that for noise exposure. In this study, we present the relationship of *L*_vzmax_ and maximum transient vibration value (*MTVV*: m/s^2^) [14] for vertical ground vibration induced by the Shinkansen railway. Shimoyama et al. [15] indicated the following approximate expression:

$$10\log_{10}{\left(MTVV/10^{-5}\right)}^{2}=Lvz\max+1.8.$$

The noise and vibration exposure values were rounded to one decimal place in this paper.

## 3. Results

### 3.1. Demographic Factors

Table 2 shows the relative frequency distributions of demographic and housing factors. Overall, there were proportionally more female respondents than male respondents. In the KNG95 dataset, the survey target of housewives accounted for this bias. Respondents aged 50 years or older accounted for 52–83% of each dataset. This may have been driven by the fact that the analysis examined only detached houses. Regarding the period of residence, more than 70% had lived continuously in their home for 10 years or more. Most of the detached houses had wooden frame structures, except for the NGY dataset. Based on lessons from disasters in the past, many inhabitants may live in detached houses made of reinforced concrete.

### 3.2. Noise and Vibration Exposures

**Figure 1:**
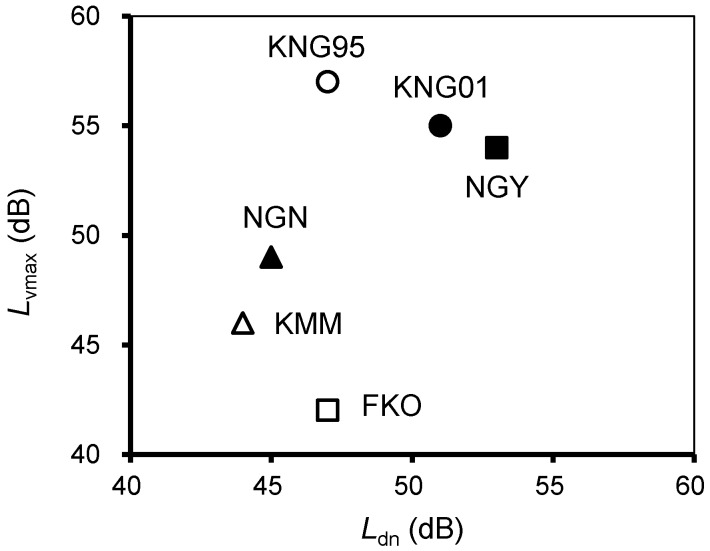
Relationships between the mean energy-based index (*L*_dn_) and mean maximum-based index (*L*_vmax_) for each dataset.

Figure 1 shows the relationships between the mean *L*_dn_ and mean *L*_vmax_ for each dataset. Since each dataset covered a different survey area, we focused on their relative positional relationships. The KNG95, KNG01 and NGY datasets indicated higher noise and vibration exposures than the others. In particular, the KNG95 dataset had the highest *L*_vmax_, whereas the *L*_dn_ of the KNG95 dataset was lower than those of the KNG01 and NGY datasets. Comparing the *L*_dn_ among the six datasets, the KMM and NGN datasets had lower values, attributed to the lower frequency of train service than that of the other lines. Similar to *L*_dn_, the *L*_vmax_ of newer Shinkansen lines, the KSL and HSL, were relatively lower. This was due to the increased weight of the railway structure and improved countermeasures against ground vibrations. Meanwhile, the FKO dataset had the lowest *L*_vmax_, possibly because of the proportion of dwellings located more than 100 m from the Shinkansen line. In the FKO dataset, 41% were located more than 100 m from the line, which was followed by the KMM (40%) and KNG95 (17%) datasets, while the other datasets were less than 10%.

### 3.3. Exposure-Response Relationships

To express exposure-response relationships, we used the %Extremely Annoyed (abbreviated as %EA) and %Very Annoyed (abbreviated as %VA) as indices of annoyance. For the five-point scale in this study, %EA is calculated from the rate of people who responded to the most annoyed category. The %VA was the rate of people who respond to either of the top two categories.

**Figure 2:**
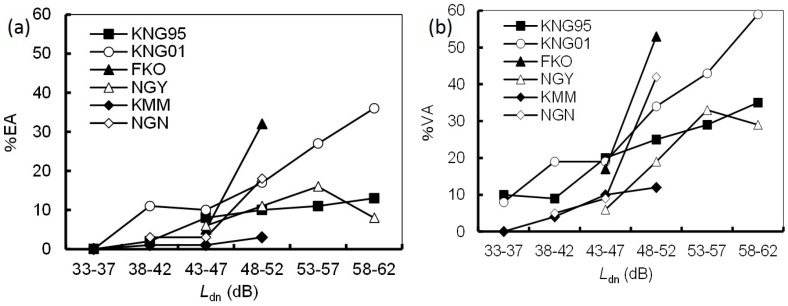
Comparison of the relationships between *L*_dn_ and prevalence of noise annoyance for each dataset. (**a**) *L*_dn_–%EA relationships; (**b**) *L*_dn_–%VA relationships. Index of annoyance calculated from the rate of people who responded to the most annoyed category (%EA); the rate of people who respond to either of the top two annoyance categories (%VA).

**Figure 3:**
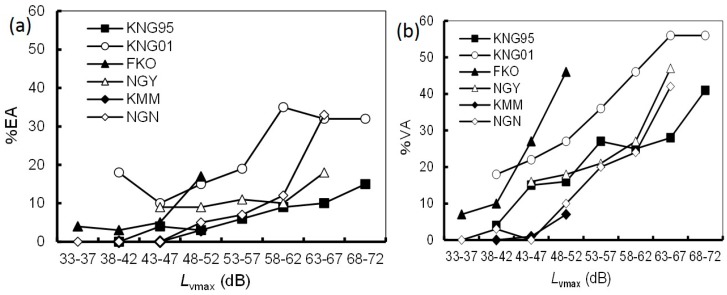
Comparison of the relationships between *L*_vmax_ and prevalence of vibration annoyance for each dataset. (**a**) *L*_vmax_–%EA relationships; (**b**) *L*_vmax_–%VA relationships.

Figure 2 shows a comparison of the relationships between *L*_dn_ and the prevalence of noise annoyance for each dataset. The *L*_dn_ range was divided into 5-dB intervals. It should be noted that %EA/%VA was not plotted for intervals containing fewer than 10 respondents. The difference in the prevalence of noise annoyance varied greatly among the datasets. The KNG01 and FKO datasets exhibited the highest *L*_dn_–%EA/%VA relationships. In particular, the *L*_dn_–%EA/%VA relationship for the FKO dataset rose rapidly in the range of 48–52 dB. In contrast, the KMM and NGN datasets showed relatively lower *L*_dn_–%EA/%VA relationships, although the NGN dataset showed a rapid rise similar to the FKO dataset. In particular, few inhabitants were annoyed by noise in the KMM dataset. Figure 3 shows a comparison of the relationships between *L*_vmax_ and the prevalence of vibration annoyance for each dataset. The *L*_vmax_ range was divided into 5-dB intervals. The *L*_vmax_–%EA relationship for the KNG01 dataset was remarkably high, whereas other datasets were relatively consistent. Similar trends were observed in the *L*_vmax_–%VA relationships.

To confirm the effect of the difference in annoyance descriptors, ICBEN and bearable scales, we focused on the difference in the exposure-response relationships between the KNG95 and KNG01 datasets, which were derived from the surveys conducted in the Kanagawa Prefecture [3]. Since it was difficult to compare exposure-response relationships directly, we used satisfaction-dissatisfaction responses to outdoor quietness and house vibrations, which were common questions in both surveys. The relationship between *L*_dn_ and the response to outdoor quietness in the KNG01 dataset was similar to, or even slightly more favorable to that in the KNG95 dataset. Likewise similar trends were confirmed for the relationship between *L*_vmax_ and the response to house vibrations. These findings were not consistent with the change in the prevalence of annoyance. Therefore, we presumed that the difference in %EA/%VA originated in the difference in the definition of annoyance. Further analysis was done excluding the KNG95 dataset, focusing only on the dataset with the ICBEN verbal scale.

Excluding the KNG95 dataset, the five included surveys represented a time scale of 10 years. Nagano and Kyushu Shinkansen lines began operation in 1997 and 2011, respectively [16]. Both lines are newer than the Tokaido (1964) and Sanyo (1972) Shinkansen lines. As shown in Figure 2 and Figure 3, the prevalence of annoyance due to noise/vibration from the new lines was relatively lower than that from the old lines. In other words, annoyance decreased over the 10 years. This finding is not consistent with previous results [17]. More effective noise and vibration countermeasures were implemented for the new Shinkansen lines than for the old Shinkansen lines. Therefore, we consider that countermeasures rather than a temporal effect resulted in under response for the new Shinkansen lines.

### 3.4. Combined Effects

**Figure 4:**
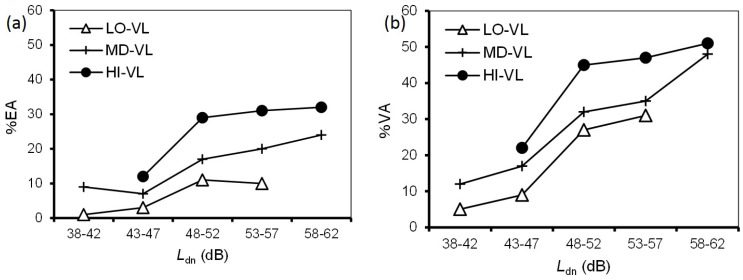
Comparison of the relationships between *L*_dn_ and prevalence of noise annoyance according to vibration exposures: (**a**) *L*_dn_–%EA relationships; (**b**) *L*_dn_–%VA relationships. Low noise level group (LO-NL); medium noise level group (MD-NL); high noise level groups (HI-NL).

**Figure 5:**
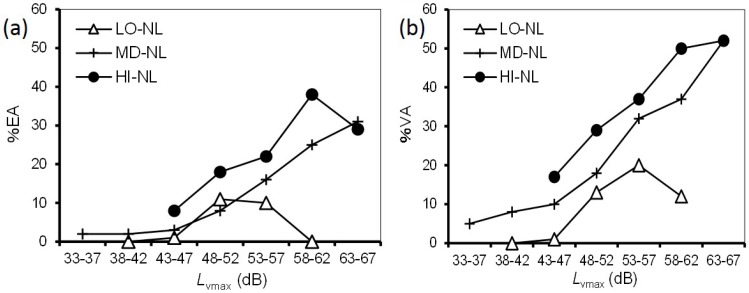
Comparison of the relationships between *L*_vmax_ and prevalence of vibration annoyance according to noise exposures: (**a**) *L*_vmax_–%EA relationships; (**b**) *L*_vmax_–%VA relationships.

We investigated the combined effects of noise and ground vibration from the Shinkansen railway on annoyance. First, we addressed the effect of ground vibration on noise annoyance. Figure 4 shows a comparison of the relationships between noise exposure and prevalence of noise annoyance according to vibration exposure. Considering sample size, respondents were divided into three groups, low, medium, and high vibration levels at 10-dB intervals: LO-VL (38–47 dB); MD-VL (48–57 dB), and; HI-VL (58–67 dB). Each group contained more than 300 respondents. %EA/%VA was not plotted for intervals where a group contained fewer than 10 respondents. The prevalence proportion in each noise exposure range increased with increasing vibration exposure (Figure 4). Comparing the differences in vibration exposure range, the difference between HI-VL and MD-VL was larger than that between MD-VL and LO-VL. Figure 5 shows a comparison of the relationships between vibration exposure and the prevalence of vibration annoyance according to noise exposures, divided into three low, medium, and high noise level groups at 10-dB intervals, LO-NL (33–42 dB), MD-NL (43–52 dB), and HI-NL (53–62 dB). The sample size of each group was greater than 200. For the *L*_vmax_–%EA relationships, HI-NL was higher than MD-NL, while there was little difference between LO-NL and MD-NL. For the *L*_vmax_–%VA relationships, the three relationships were located at relatively equal intervals.

### 3.5. Application of Logistic Regression Analysis

To quantify the combined effects of noise and vibration on annoyance, we applied logistic regression analysis to the datasets. The analysis was applied only to extremely and very annoyed responses due to noise and ground vibration as the dependent variable, while exposure levels (*L*_dn_ and *L*_vmax_), sex, age, and housing structure were included as independent variables. Noise and vibration exposures were considered as category scales in 5- or 10-dB intervals. When applied to noise annoyance, five categories in 5-dB intervals of *L*_dn_ (38–62 dB) and three categories of *L*_vmax_ (LO-VL, MD-VL, and HI-VL) were used as the exposure levels. When analyzing vibration annoyance, seven categories in 5-dB intervals of *L*_vmax_ (33–67 dB) and three categories of *L*_dn_ (LO-NL, MD-NL, and HI-NL) were used as the exposure levels.

Table 3 and Table 4 show the odds ratio of each category for the noise and vibration annoyances, respectively. Since the noise and vibration annoyance models with the interaction of noise and vibration exposures, to which we applied logistic regression analysis, were not significant, we showed the result without the interaction. The odds ratios for noise annoyance were based on the following reference categories: male (sex), <30 (age), wood (housing structure), 38–42 dB (*L*_dn_), and MD-VL (*L*_vmax_). For the vibration annoyance, 33–37 dB (*L*_vmax_) and MD-NL (*L*_dn_) were set as the reference vibration and noise exposures, respectively.

As shown in Table 3, within an *L*_dn_ range of 48–52 dB, the odds ratio was much larger than 1 and the lower limit of the 95% confidence interval was also above 1. For the LO-VL category, the odds ratio and the upper limit of the 95% confidence interval were less than 1. HI-VL was significantly higher at the 5% level than MD-VL. These results show a significant increase in noise annoyance in the *L*_dn_ range of 48–52 dB or above and a significant effect of *L*_vmax_ on noise annoyance. Table 4 shows the same trend as that observed in Table 3, namely, a significant increase in vibration annoyance in an *L*_vmax_ range of 48–52 dB, and a significant effect of *L*_dn_ on vibration annoyance. Furthermore, females were less annoyed by noise and ground vibration than males. Age and housing structure had no significant impact on noise and vibration annoyances; however, inhabitants had a tendency to be slightly more annoyed by noise and ground vibrations in wooden detached houses than in steel framed or reinforced concrete detached houses.

**Figure 6:**
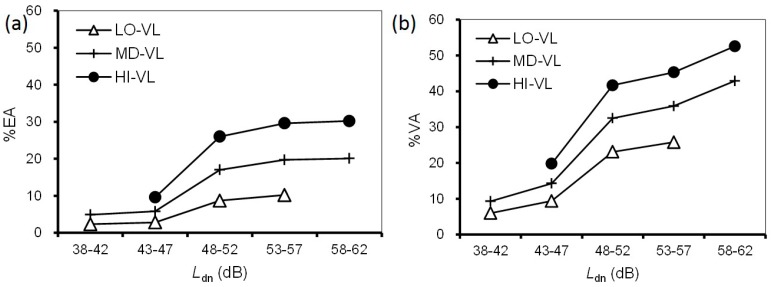
Relationships between *L*_dn_ and prevalence of noise annoyance by vibration exposures: (**a**) *L*_dn_–%EA relationships; (**b**) *L*_dn_–%VA relationships.

**Figure 7:**
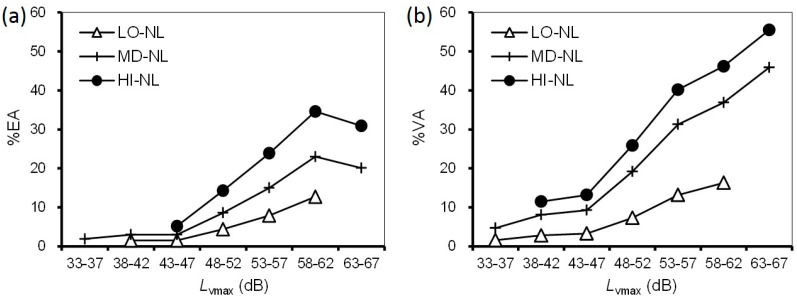
Relationships between *L*_vmax_ and prevalence of vibration annoyance by noise exposures: (**a**) *L*_vmax_–%EA relationships; (**b**) *L*_vmax_–%VA relationships.

From the results shown in Table 3 and Table 4, we developed the relationships between exposures, *L*_dn_ and *L*_vmax_, and noise/vibration annoyance. To determine each prevalence proportion, we adjusted sex and age by the results of the national census in 2015 and set wooden house as the housing structure item. The *L*_dn_–%EA/%VA relationships with different vibration exposures and *L*_vmax_–%EA/%VA relationships with different noise exposures are shown in Figure 6 and Figure 7, respectively.

Although the average slopes of the relationships between *L*_dn_ and the prevalence of noise annoyance in the range of 48–52 dB or above were moderate, particularly for %EA, the prevalence proportion noticeably increased with increasing *L*_vmax_ in every *L*_dn_ range (Figure 6). In the *L*_dn_ range of 48–52 dB, including the averaged value 48 dB of noise exposure, the prevalence proportions were 9% (LO-VL), 17% (MD-VL), and 26% (HI-VL) for %EA and 23% (LO-VL), 33% (MD-NL), and 42% (HI-VL) for %VA. Likewise, the prevalence proportion gradually increased with increasing vibration exposure, except in the range of 63–67 dB (Figure 7). Meanwhile, the exposure-response relationships increased with increasing noise exposure at the same vibration exposure. The difference in the *L*_vmax_–%VA relationships between LO-NL and MD-NL was larger than that between MD-NL and HI-NL, particularly for %VA in the range of 48–52 dB or higher. For example, in the *L*_vmax_ range of 48–52 dB, including the averaged value 50 dB of vibration exposure, the prevalence proportions were 5% (LO-NL), 9% (MD-NL), and 14% (HI-NL) for %EA and 7% (LO-NL), 19% (MD-NL), and 26% (HI-NL) for %VA.

These observations confirmed that, as vibration and noise exposure increased, the associated noise and vibration annoyances also increased, respectively, particularly at medium and high levels of both exposures. The *L*_dn_–%EA/%VA relationships among the three categories of *L*_vmax_ showed an increase with increasing *L*_vmax_. Likewise, the *L*_vmax_–%EA/%VA relationships according to three categories of *L*_dn_ showed similar trends with increasing *L*_dn_. These results suggest a combined effect of noise and vibration exposures on annoyance when inhabitants are exposed to higher exposure levels of noise and vibration.

## 4. Discussion

Based on a reanalysis of datasets compiled in six socio-acoustic surveys conducted separately over the last 20 years in Japan, we investigated the combined effects of noise and ground vibration on annoyance of inhabitants living near the Shinkansen railway lines. In this study, we confirmed the combined effect of noise and vibration exposure on annoyance. However, Lee et al. [12] revealed that vibration did not influence noise annoyance in a laboratory-based experiment. Several laboratory studies that examined the combined effects of noise and vibration on annoyance also reported similar findings [18,19]. To discuss the differences in the results of combined effects on annoyance between field studies and laboratory experiments, we considered the differences in the circumstances in which subjects were exposed to noise and vibration, as referenced by Lee et al. [12] and Gidlöf-Gunnarsson et al. [20]. Inhabitants in detached houses targeted for social surveys are frequently exposed to noise and vibration simultaneously over a long period of time. Öhrström [21] compared conventional railway noise annoyance between areas with and without vibration and showed that noise annoyance was 10 dB severer in areas with strong vibration than those without vibration. This is consistent with our finding that difference in the %VA due to noise between HI-VL and MD-VL corresponded to about 10 dB of noise level in the *L*_dn_ range of 48–52 dB or above. Lercher [22] reviewed studies on combined effects of noise and vibration and summarized that vibration increased noise annoyance, although the level-dependency was different among the studies. In addition, Tamura [23] found that noise from the Shinkansen railway was regarded more negatively than conventional railway noise. He also indicated that inhabitants living in the areas along the Shinkansen railway were generally concerned with noise issues and recognized no need for the Shinkansen railway. Under such circumstances, it can be assumed that there is a combined effect on annoyance, particularly at higher exposure levels. In contrast, the subjects of laboratory experiments are only exposed to noise and vibration during the study, and it may be more difficult to reproduce the combined effect on annoyance, unlike in actual living environments that are affected by a variety of factors.

Next, we estimated the vibration exposure based on vertical measurements obtained on the ground surface. However, even using vertical measurements, house vibrations differ from ground vibrations. Therefore, these measurements could not provide exact estimates of the vibration exposure to inhabitants in their detached houses. Regardless, according to a recent study concerning the vibration amplitude in wooden or steel detached houses, there was little difference in the vertical vibration between the ground and floors [24]. Therefore, since the vibration exposure was divided into 5-dB intervals in this study, we expect that the average exposure-response relationships proposed in this study provide good approximations of vertical vibration exposure. However, horizontal vibrations, which are amplified in detached houses at resonance frequencies, were not estimated. In recent years, complaints and problems induced by horizontal vibrations have been reported [25]. As such, further research is required to establish a prediction method for vertical and horizontal vibration exposures in wooden and steel detached houses.

Then, the prevalence of noise/vibration annoyances was calculated based on 5- and 10-dB intervals of individual exposures. Because of some of the problems mentioned above, these intervals were satisfactory, and smaller intervals (e.g., 1-dB intervals) would likely not have provided additional information for vibration exposure. Regarding noise exposure, considering the accuracy of the estimation methods, we believe that the use of 5-dB intervals provides meaningful results. Moreover, the use of 10-dB intervals is also practical given current constraints and provides sufficient information; however, 5-dB intervals could provide more effective technical material. Future research on annoyance should establish methods to meaningfully divide noise and vibration data into smaller intervals to provide more accurate results that can be used by railway designers and policymakers.

As shown in Figure 6 and Figure 7, though the prevalence of noise annoyance was saturated in the *L*_dn_ range of 48–52 dB, the prevalence of vibration annoyance continuously increased with vibration exposure, particularly for %VA. At higher levels of noise or vibration exposure, the majority of respondents were those of the KNG01 survey. Presumably, inhabitants have lived with high noise exposure levels for a long time could tolerate the adverse effects on their living environment. Meanwhile, the difference in the prevalence of noise and vibration annoyances suggests that vibration causes greater annoyance in inhabitants than noise. This tendency was consistent with the greater effect of vibration exposure than noise exposure on the combined annoyance due to noise and vibration from the Tokaido Shinkansen railway [1].

Finally, considering the inhabitants’ perspective, it is important to develop an evaluation method of the concept of “combined annoyance” due to noise and ground-borne vibration. However, noise/vibration annoyance is a multifaceted psychological construct that is affected by many other factors, including visibility, attitude towards the source, demographic factors, and so forth [26]. In addition, noise and vibration annoyances affect each other. Thus, developing an evaluation method is indispensable for discussing non-acoustical and non-vibrational factors affecting individual annoyance. Therefore, the previous findings were reviewed to determine which factors affected. Regarding the visual effect, Maffei et al. [27] observed that the perceived loudness and noise annoyance derived from railway noise differed between visible and non-visible trains. Similarly, Peris et al. [28] studied the impact of the visibility of a railway on vibration annoyance and found that, at the same vibration exposure level, the odds ratio of people being highly annoyed by vibration from the railway was much higher when the railway was visible than when it was not visible. However, since many of the datasets used for this study did not include a response on the visibility of the target trains, this effect could not be analyzed fully. Regarding the attitude towards the source, Tamura [23], Sato [29], and Pedersen et al. [30], reported that attitudes were associated with noise annoyance. A comparison of the exposure-annoyance relationships for noise and vibration suggested that vibration annoyance could be affected more by these factors than noise annoyance at higher exposure levels. In addition, sex and age had characteristic effects on noise/vibration annoyance in this analysis. Regarding sex, males were significantly more annoyed by noise/vibration than females. Although age had no significant effect on noise/vibration annoyance, the observed pattern was consistent with previous findings of a curvilinear effect, as indicated by van Gervan et al. [31].

## 5. Conclusions

We performed a reanalysis of individual datasets of exposure and community response to noise and vibration generated by the Shinkansen railway. The data were compiled in six socio-acoustic surveys, conducted separately over the past 20 years in Japan. Applying logistic regression analysis to the datasets, we investigated the combined effects of vibration exposure on noise annoyance and noise exposure on vibration annoyance. The significantly increasing effect of noise and vibration on vibration and noise annoyances confirmed the presence of combined effects of both exposures on annoyance. Based on the results, we proposed a representative relationship between noise and vibration exposures, and the prevalence of each annoyance associated with the Shinkansen railway. In future research, we intend to address the combined effects of noise and vibration exposures on annoyance from conventional railway. Moreover, through the comparison between community responses to conventional railway and high-speed railway in Japan, we would like to clarify the difference in the degree of combined effects due to conventional railway among Japan, other Asian and Western countries.

## Figures and Tables

**Table 1:** Outline of datasets.

Dataset ^a^	KNG95	KNG01	FKO	NGY	KMM	NGN
Date (FY)	1995–1996	2001–2003	2003	2004	2011–2012	2013
Location ^b^	KNG Pref.	KNG Pref.	FKO Pref.	NGY City	KMM Pref.	NGN Pref.
Line ^c^	TSL	TSL	SSL	TSL	KSL	HSL
Number of trains ^d^						
Daytime (7:00–22:00)	234	265	142	249	112	62
Nighttime (22:00–7:00)	16	22	18	21	17	7
Method	Visit-Mail ^e^	Visit-Mail ^e^	Visit-Mail ^e^	Interview	Visit-Visit ^e^	Mail-Mail ^e^
Housing type (DH; AB) ^f^	DH	DH & AB	DH	DH	DH & AB	DH
Annoyance	unbearable	ICBEN ^g^	ICBEN ^g^	ICBEN ^g^	ICBEN ^g^	ICBEN ^g^
Response proportion (%) ^h^	72	57	66	58	45	45
Sample size	709	872	358	175	559	294

^a^ References for datasets (left–right): (3, 1, 2, 5, 10, 8); ^b^ Kanagawa (KNG); Fukuoka (FKO); Nagoya (NGY); Kumamoto (KMM), and; Nagano (NGN); ^c^ Tokaido Shinkansen line (TSL); Sanyo Shinkansen line (SSL); Hokuriku Shinkansen line (HSL); Kyushu Shinkansen line (KSL); ^d^ Shinkansen super-express trains operate between 6:00 and 0:00; ^e^ Distribution-collection method; ^f^ Detached house (DH); Apartment building (AB); ^g^ International Commission on Biological Effects of Noise (ICBEN); ^h^ Response proportion of detached houses.

**Table 2:** Relative frequency of distributions of demographic and housing factors (%).

Items	Categories	KNG95	KNG01	FKO	NGY	KMM	NGN
Gender	Male	14	47	49	39	41	56
	Female	86	50	50	61	58	35
	Unknown	1	3	0	1	1	10
Age	<30	6	3	7	4	4	3
	30–39	10	12	7	5	4	4
	40–49	31	17	10	10	12	9
	50–59	30	29	24	16	17	16
	60–69	17	22	37	31	28	33
	≥70	4	15	15	33	33	34
	Unknown	0	2	0	1	2	1
Period of residence	<5 years	14	17	5	9	6	4
	<10 years	12	13	9	5	11	10
	≥10 years	74	69	86	83	83	85
	Unknown	0	0	0	3	1	1
Housing structure	Wooden	85	86	91	59	89	86
	Reinforced Concrete	1	2	1	23	3	2
	Steel frame	10	10	6	7	7	10
	Others	3	0	1	6	0	1
	Unknown	1	2	0	4	1	0

**Table 3:** Logistic regression analysis of %EA/%VA due to noise.

Item	Category	%EA		%VA	
		Odds Ratio	95% CI	Odds Ratio	95% CI
*L*_dn_	43–47	1.204	0.542–2.676	1.627	0.938–2.823
	48–52	3.988	1.889–8.421	4.704	2.762–8.014
	53–57	4.780	2.171–10.524	5.459	3.062–9.732
	58–62	4.922	2.026–11.957	7.319	3.689–14.519
*L*_vmax_	LO-VL	0.466	0.310–0.700	0.621	0.471–0.820
	HI-VL	1.717	1.215–2.426	1.482	1.104–1.989
Sex	Female	0.680	0.507–0.910	0.719	0.573–0.903
Age	30–39	1.287	0.506–3.271	0.770	0.389–1.521
	40–49	1.420	0.585–3.448	1.039	0.554–1.951
	50–59	1.708	0.733–3.979	1.254	0.692–2.272
	60–69	1.600	0.688–3.723	1.324	0.734–2.387
	≥70	0.751	0.308–1.832	0.604	0.325–1.120
Housing Structure	Reinforced Concrete	0.577	0.267–1.248	0.734	0.419–1.285
	Steel frame	0.882	0.521–1.491	1.044	0.705–1.547
	Others	0.529	0.119–2.342	0.487	0.160–1.478
Constant		0.051		0.130	

**Table 4:** Logistic regression analysis of %EA/%VA due to ground vibration.

Item	Category	%EA		%VA	
		Odds Ratio	95% CI	Odds Ratio	95% CI
*L*_vmax_	38–42	1.640	0.324–8.305	1.778	0.629–5.029
	43–47	1.625	0.367–7.201	2.084	0.805–5.398
	48–52	4.923	1.166–20.833	4.804	1.884–12.250
	53–57	9.277	2.220–38.778	9.231	3.6381–23.422
	58–62	15.659	3.709–66.111	11.804	4.569–30.498
	63–67	13.215	2.982–58.557	17.126	6.261–46.843
*L*_dn_	LO-NL	0.489	0.248–0.963	0.333	0.196–0.565
	HI-NL	1.775	1.259–2.502	1.470	1.099–1.968
Sex	Female	0.721	0.535–0.972	0.621	0.490–0.786
Age	30–39	0.887	0.353–2.231	0.720	0.359–1.443
	40–49	1.379	0.589–3.231	1.157	0.611–2.189
	50–59	1.706	0.761–3.826	1.304	0.714–2.383
	60–69	1.126	0.498–2.547	0.982	0.537–1.794
	≥70	0.824	0.352–1.929	0.608	0.324–1.140
Housing Structure	Reinforced Concrete	0.748	0.355–1.576	0.814	0.455–1.456
	Steel frame	0.842	0.491–1.443	0.863	0.567–1.314
	Others	0.598	0.130–2.736	1.450	0.562–3.742
Constant		0.020		0.070	
